# Supplementary material for: Retinoic Acid Receptor Alpha (RARα) in Macrophages Protects from Diet-Induced Atherosclerosis in Mice
Source: Cells. 2022 Oct 11;11(20):3186. doi: 10.3390/cells11203186 (PMC9600071; doi:10.3390/cells11203186)
Supplement: Supplementary file 1 [file cells-11-03186-s001.zip › cells-1942024-supplementary.pdf]

Supplementary Information

Retinoic Acid Receptor Alpha (RARα) in Macrophages Protects from Diet-induced Atherosclerosis in Mice

Fathima N. Cassim Bawa, Raja Gopoju, Yanyong Xu, Shuwei Hu, Yingdong Zhu, Shaoru Chen, Kavita Jadhav, Yanqiao Zhang

Table S1. qRT-PCR primer sequences

Genes	Primer sequences
36b4	Forward sequence: 5’-GGCCCGAGAAGACCTCCTT<br>Reverse sequence: 5’-TCAATGGTGCCTCTGGAGATT
Abca1	Forward sequence: 5’-OGTTTCCGGGAAGTGTCCTA<br>Reverse sequence: 5’-CTAGAGATGACAAGGAGGATGGA
Abcg1	Forward sequence: 5’-CCTTCCTCAGCATCATGCG<br>Reverse sequence: 5’-CCGATCCCAATGTGCGA
Il-1β	Forward sequence: 5’-CCTGAACTCAACTGTGAAATGC<br>Reverse sequence: 5’-GCGAGATTTGAAGCTGGATG
Il-6	Forward sequence: 5’-GAGACTTCACAGAGGATACCAC<br>Reverse sequence: 5’-TCAGAATTGCCATTGCACAAC
Rarα	Forward sequence: 5’-CTGTGAGGGCTGTAAGGG<br>Reverse sequence: 5’-TGATGCAGTTCTTGTCCCG
Tnfα	Forward sequence: 5’-CCCTCCAGAAAAGACACCATG<br>Reverse sequence: 5’-GCCACAAGCAGGAATGAGAAG

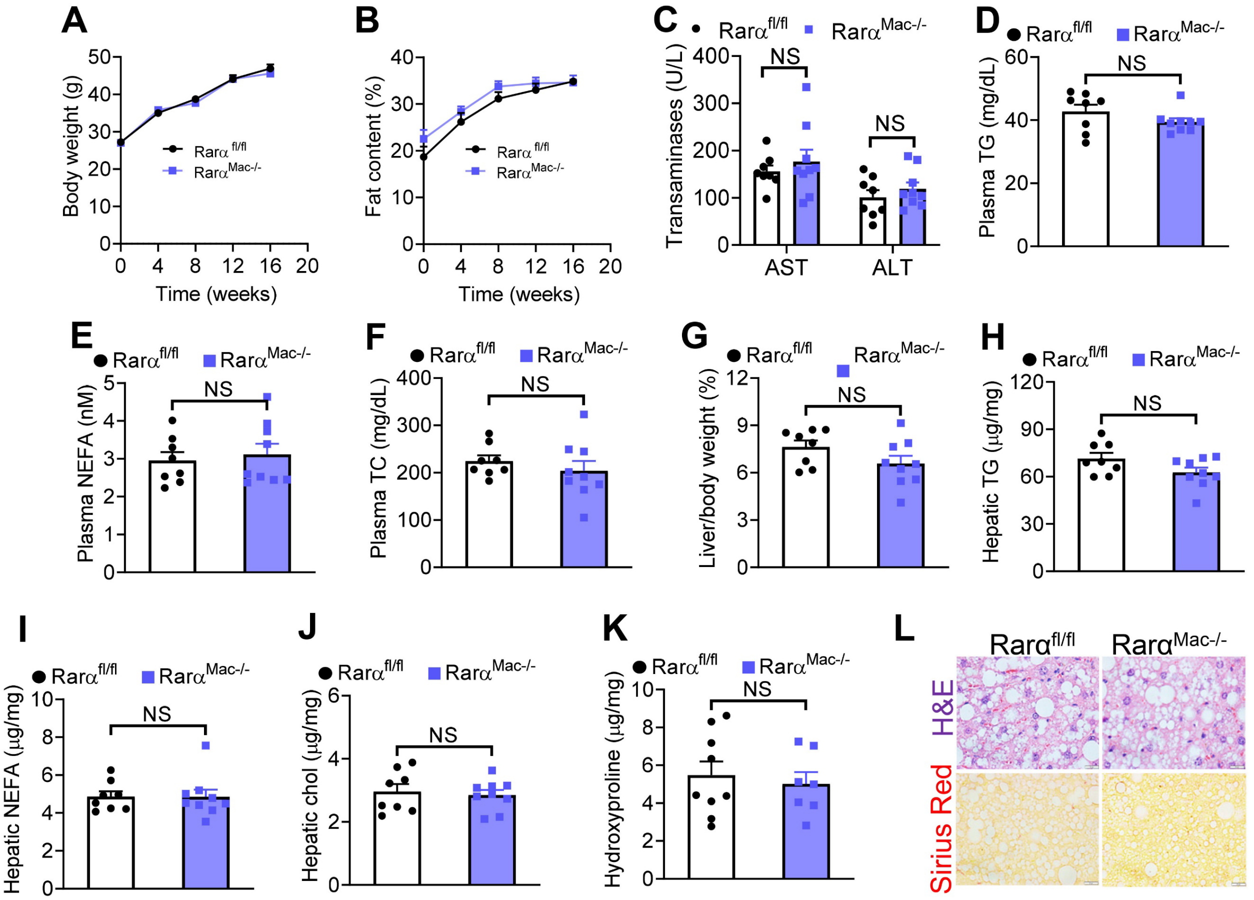

Figure S1. Loss of RARα in macrophages does not affect body weight gain, body fat content, plasma or hepatic lipids, or fibrogenesis in Western diet-fed mice

Rarα$^{fl/fl}$ or Rarα$^{Mac-/-}$ mice were fed an HFHC diet for 16 weeks (n=8-9). Body weight gain (**A**) and body fat content (**B**) were measured. Plasma levels of AST and ALT (**C**), TG (**D**), NEFA (**E**) and total cholesterol (**F**) were determined. Liver weight to body weight ratio (%) was measured (**G**). Hepatic TG (**H**), NEFA (**I**), total cholesterol (**J**), and hydroxyproline (**K**) levels were measured. Representative liver images of H&E staining (upper panels) and picrosirius red staining (lower panels) are presented (**L**) (scale bars = 20 μm). All the data are expressed as mean±SEM. NS, not significant

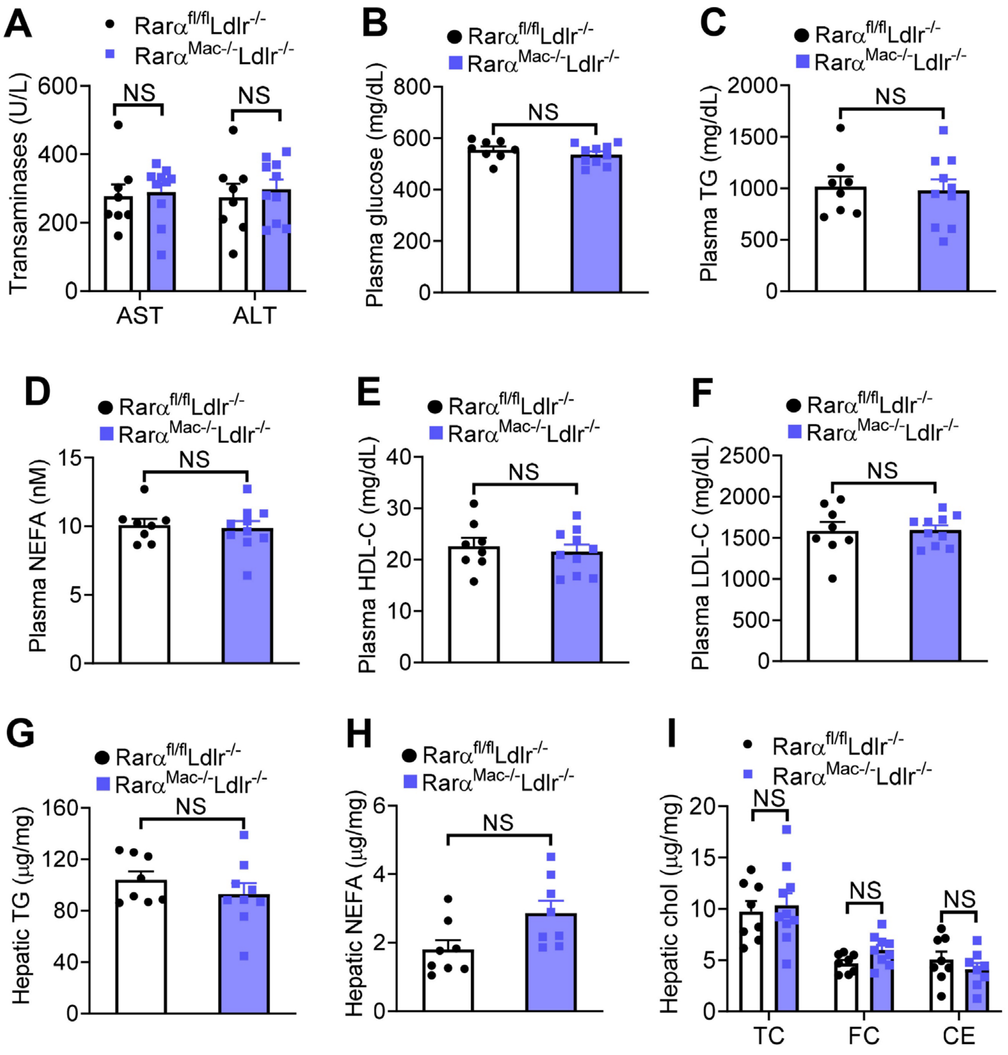

Figure S2. Loss of RARα in macrophages does not regulate plasma or hepatic lipids in Western diet-fed *Ldlr*$^{-/-}$ mice

Rarα$^{fl/fl}$*Ldlr*$^{-/-}$ and *Rarα*$^{Mac-/-}$*Ldlr*$^{-/-}$ mice were fed a Western diet for 16 weeks (n=8-9). Plasma AST and ALT (**A**), glucose (**B**), TG (**C**), NEFA (**D**), HDL-C (**E**), and LDL-C (**F**) levels were determined. Hepatic TG (**G**), NEFA (**H**), and cholesterol (**I**) levels were determined. All the data are expressed as mean±SEM. NS, not significant
